# Constipation and the Corset

**Published:** 1898-01-22

**Authors:** 


### CONSTIPATION AND THE CORSET.

In a clinical lecture recently delivered by Dr. Seymour Taylor on Some Common Ailments, an interesting observation is made in regard to what is perhaps the commonest of all the ailments from which modern woman suffers—namely, constipation. Dr. Taylor has no doubt that this is largely due to their method of dress. The corset is not a compressor of the chest so much as of the abdomen, and this continuous pressure retards that peristaltic action which is necessary if a daily action of the bowels is to be secured. The worst cases of constipation which he has met with have been amongst the young ladies who have to study their figure and their appearance, such as those who are working in milliners' shops, as well as among ladies in the higher walks of life. Rich people, especially young people, do not suffer from want of exercise. They have horse riding and plenty of games, yet the womenkind suffer greatly from constipation, the reason in the majority of cases being the corset. This appliance would also seem to be the cause of the pain in the flanks which is so often complained of. The course of the colon is distorted, so that flatus accumulates and causes much distress. Giving up the use of the corset, then, is likely to add greatly to the efficacy of any treatment which may be recommended for the constipation. Now, as regards treatment, Dr. Taylor recommends castor oil in small doses, and says that it is not necessary in ordinary practice to prescribe a more severe remedy. By giving a drachm of castor oil, fasting, the bowel may be coaxed into its proper action; but the oil must be continued for several weeks. If this does not succeed, a small quantity of croton oil may be added to the castor oil, say one-sixth or one-quarter of a drop in each drachm. Dr. Taylor then goes on to give many details about the general treatment of constipation, but the matters to which alone we here wish to draw attention are the causation of constipation by the corset, and its treatment by small repeated doses of castor oil—a proceeding which is to a large extent shorn of its terrors when once the art of swallowing capsules has been attained.

